# Hotspot autoimmune T cell receptor binding underlies pathogen and insulin peptide cross-reactivity

**DOI:** 10.1172/JCI85679

**Published:** 2016-05-16

**Authors:** David K. Cole, Anna M. Bulek, Garry Dolton, Andrea J. Schauenberg, Barbara Szomolay, William Rittase, Andrew Trimby, Prithiviraj Jothikumar, Anna Fuller, Ania Skowera, Jamie Rossjohn, Cheng Zhu, John J. Miles, Mark Peakman, Linda Wooldridge, Pierre J. Rizkallah, Andrew K. Sewell

**Affiliations:** 1 Division of Infection and Immunity and Systems Immunity Research Institute, Cardiff University School of Medicine, Heath Park, Cardiff, United Kingdom.; 2 Mathematics Institute, University of Warwick, Coventry, United Kingdom.; 3 Woodruff School of Mechanical Engineering and Coulter Department of Biomedical Engineering, Georgia Institute of Technology, Atlanta, Georgia, USA.; 4 Department of Immunobiology, King’s College London, London, United Kingdom.; 5 NIHR Biomedical Research Centre at Guy’s and St Thomas’ NHS Foundation Trust and King’s College London, London, United Kingdom.; 6 Department of Biochemistry and Molecular Biology, School of Biomedical Sciences, and; 7 ARC Centre of Excellence in Advanced Molecular Imaging, Monash University, Clayton, Victoria, Australia.; 8 Coulter Department of Biomedical Engineering, Georgia Institute of Technology, Atlanta, Georgia, USA.; 9 QIMR Berghofer Medical Research Institute, Brisbane, Queensland, Australia.; 10 Faculty of Health Sciences, University of Bristol, Bristol, United Kingdom.

## Abstract

The cross-reactivity of T cells with pathogen- and self-derived peptides has been implicated as a pathway involved in the development of autoimmunity. However, the mechanisms that allow the clonal T cell antigen receptor (TCR) to functionally engage multiple peptide–major histocompatibility complexes (pMHC) are unclear. Here, we studied multiligand discrimination by a human, preproinsulin reactive, MHC class-I–restricted CD8^+^ T cell clone (1E6) that can recognize over 1 million different peptides. We generated high-resolution structures of the 1E6 TCR bound to 7 altered peptide ligands, including a pathogen-derived peptide that was an order of magnitude more potent than the natural self-peptide. Evaluation of these structures demonstrated that binding was stabilized through a conserved lock-and-key–like minimal binding footprint that enables 1E6 TCR to tolerate vast numbers of substitutions outside of this so-called hotspot. Highly potent antigens of the 1E6 TCR engaged with a strong antipathogen-like binding affinity; this engagement was governed though an energetic switch from an enthalpically to entropically driven interaction compared with the natural autoimmune ligand. Together, these data highlight how T cell cross-reactivity with pathogen-derived antigens might break self-tolerance to induce autoimmune disease.

## Introduction

T cells perform an essential role in adaptive immunity by interrogating the host proteome for anomalies, classically by recognizing peptides bound in major histocompatibility (MHC) molecules at the cell surface. Recent data (1–3) supports the notion that, to perform this role, the highly variable αβ T cell antigen receptor (TCR) must be able to recognize thousands, if not millions, of different peptide ligands (4, 5). This ability is required to enable the estimated 25 million distinct TCRs expressed in humans (6) to provide effective immune coverage against all possible foreign peptide antigens (5). Although essential to avoid blind spots during pathogen recognition, T cell cross-reactivity has also been implicated as a pathway to autoimmunity, possibly mediated by highly reactive pathogen-specific T cells weakly recognizing self-ligands (7–10).

Several mechanisms, by which TCRs could bind to a large number of different peptide-MHC (pMHC), have been proposed (5). Structures of unligated and ligated TCRs have shown that the TCR complementarity determining region (CDR) loops can be flexible, perhaps enabling peptide binding using different loop conformations (11, 12). Both MHC and peptide have also been shown to undergo structural changes upon TCR binding, mediating an induced fit between the TCR and pMHC (13–16). Other studies, mainly in the murine system, have demonstrated that the same TCR can interact with different pMHCs using a common (1, 11, 14, 17–20) or divergent modality (21). Recent studies in model murine systems demonstrate that TCR cross-reactivity can be governed by recognition of a conserved region in the peptide that allows tolerance of peptide sequence variation outside of this hotspot (1, 22).

We recently reported that the 1E6 human CD8^+^ T cell clone — which mediates the destruction of β cells through the recognition of a major, HLA-A*0201–restricted, preproinsulin signal peptide (ALWGPDPAAA_15–24_) (23–25) — can recognize upwards of 1 million different peptides (3). CD8^+^ T cells that recognize HLA-A*0201–ALWGPDPAAA have been shown to populate insulitic lesions in patients with type 1 diabetes (T1D) (26). We demonstrated that the TCR from the 1E6 T cell clone bound to HLA-A*0201–ALWGPDPAAA using a limited footprint and very weak binding affinity (23). This first experimental evidence of a high level of CD8^+^ T cell cross-reactivity in a human autoimmune disease system hinted toward molecular mimicry by a more potent pathogenic peptide as a potential mechanism leading to β cell destruction (8, 24). Here, we solved the structure of the 1E6 TCR with 7 altered peptide ligands (APLs) determined by our previously published combinatorial peptide library (CPL) screening (3), 2 of which mapped within human pathogens. These APLs differed from the natural preproinsulin peptide by up to 7 of 10 residues. We also solved the structure of each unligated APL to investigate whether structural changes occurred before or after binding — which, combined with an in-depth cellular and biophysical analysis of the 1E6 interaction with each APL, demonstrated the molecular mechanism mediating the high level of cross-reactivity exhibited by this preproinsulin-reactive human CD8^+^ T cell clone.

## Results

### The 1E6 T cell clone recognizes APLs across a large dynamic range.

**Figure 1:**
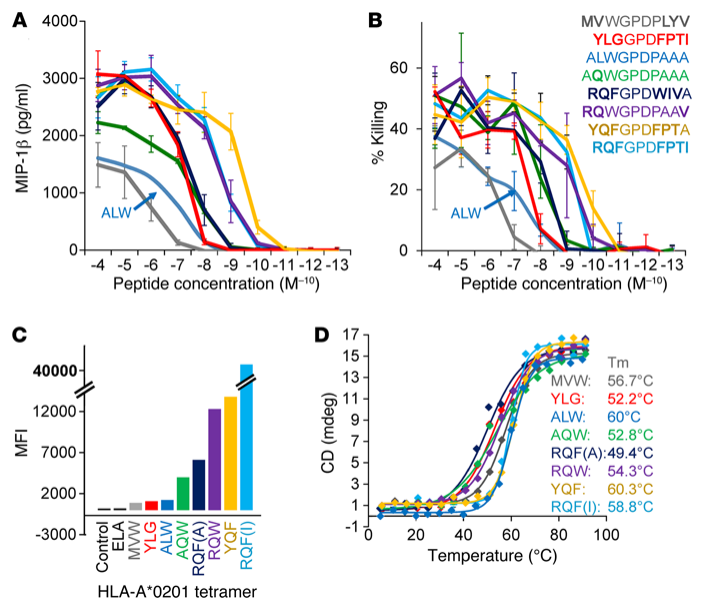
The 1E6 T cell clone reacts with a broad sensitivity range to APLs. (**A** and **B**) The 1E6 T cell clone was tested in a peptide dilution assay, in triplicate, with **MV**WGPDP**LYV** (gray), **YLG**GPD**FPTI** (red), ALWGPDPAAA (blue), A**Q**WGPDPAAA (green), **RQF**GPD**WIV**A (dark blue), **RQ**WGPDPAA**V** (purple), **YQF**GPD**FPT**A (yellow), and **RQF**GPD**FPTI** (cyan) peptides presented by HLA-A*0201–expressing C1R cells for release of MIP-1β (**A**) and killing (**B**). (**C**) The 1E6 T cell clone was stained, in duplicate, with tetramers composed of each APL (colored as above) presented by HLA-A*0201. (**D**) The stability of each APL (colored as above) was tested, in duplicate, using CD by recording the peak at 218 nm absorbance from 5°C–90°C. T_m_ values were calculated using a Boltzmann fit to each set of data.

We have previously demonstrated that the 1E6 T cell clone can recognize over 1 million different peptides with a potency comparable with, or better than, the cognate preproinsulin peptide ALWGPDPAAA (3). From this large functional scan, we selected 7 different APLs that activated the 1E6 T cell clone across a wide (4-log) functional range (Table 1). Two of these peptides, **MV**WGPDP**LYV** and **RQF**GPD**WIV**A (bold text signifies amino acids that are different from the index preproinsulin–derived sequence), are contained within the proteomes of the human pathogens *Bacteroides* *fragilis/thetaiotaomicron* and *Clostridium asparagiforme*, respectively. Competitive functional testing revealed that the preproinsulin-derived sequence ALWGPDPAAA was one of the least potent targets for 1E6, with only the **MV**WGPDP**LYV** and **YLG**GPD**FPTI** demonstrating a similar low-activity profile in MIP-1β secretion and target killing assays (Figure 1, A and B). The **RQF**GPD**WIV**A sequence (present in *C. asparagiforme*) activated the 1E6 T cell with around 1 log–greater potency compared with ALWGPDPAAA. At the other end of the spectrum, the **RQF**GPD**FPTI** peptide stimulated MIP-1β release and killing by 1E6 at an exogenous peptide concentration 2–3 logs lower compared with ALWGPDPAAA. The pattern of peptide potency was closely mirrored by pMHC tetramer staining experiments (Figure 1C and plots shown in Supplemental Figure 1; supplemental material available online with this article; doi:10.1172/JCI85679DS1). Here, the A2-**RQF**GPD**FPTI** tetramer stained 1E6 with the greatest MFI, gradually decreasing to the weakest tetramers: A2-**MV**WGPDP**LYV** and -**YLG**GPD**FPTI**. To parallel the functional analysis, we also performed thermal melt (T_m_) experiments using synchrotron radiation circular dichroism (SRCD) to investigate the stability of each APL (Figure 1D). The range of T_m_ was between 49.4°C (**RQF**GPD**WIV**A) and 60.3°C (**YQF**GPD**FPI**A), with an average approximately 55°C, similar to our previous findings (27). This pattern of stability did not correlate with the T cell activation or tetramer staining experiments, indicating that peptide binding to the MHC do not explain ligand potency.

### The 1E6 TCR can bind peptides with strong antipathogen-like affinities.

**Figure 2:**
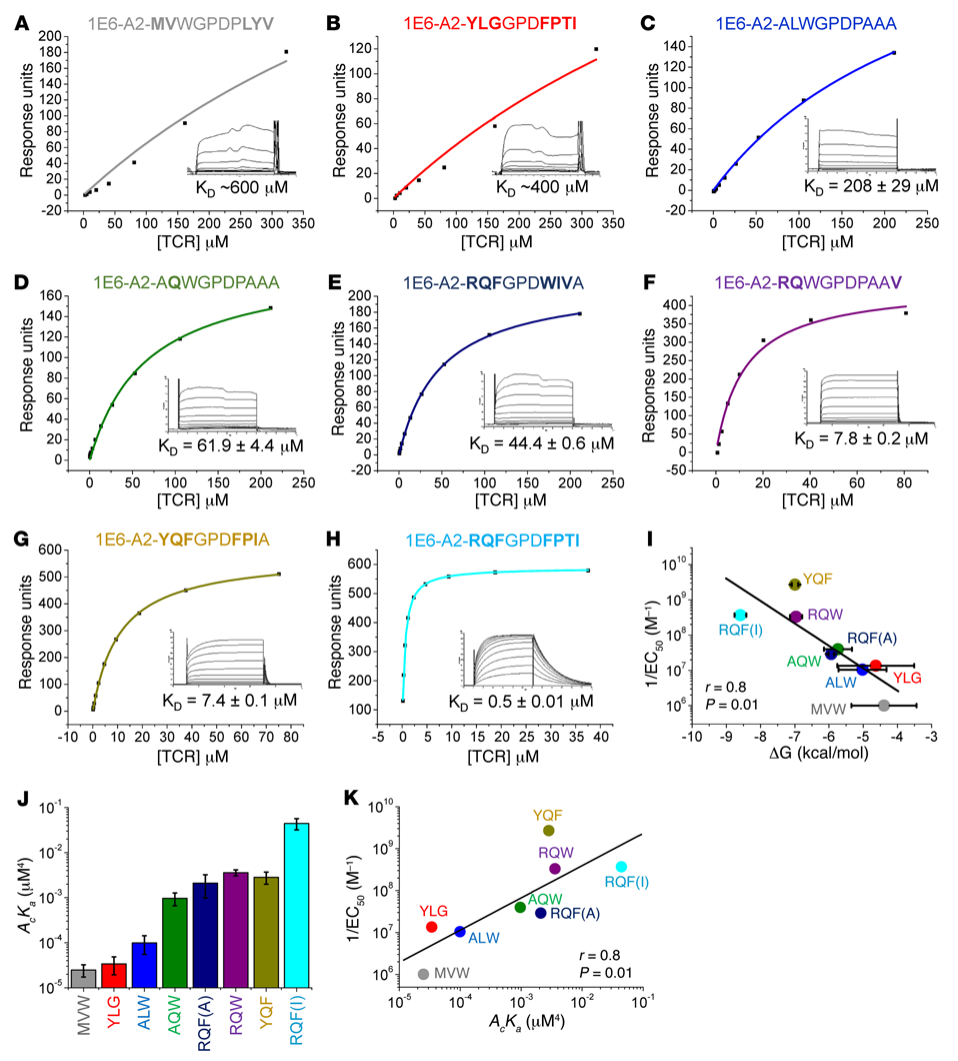
3D and 2D binding analysis of the 1E6 TCR with A2-ALW and the APLs. (**A**–**H**) Binding affinity of the 1E6 TCR interaction at 25°C using SPR. Eight serial dilutions of the 1E6 TCR were measured (shown in the inset); representative data from 3 independent experiments are plotted. The equilibrium binding constant (K_D_) values were calculated using a nonlinear curve fit (*y*= [P_1_*x*]/[P_2_ + X]). In order to calculate each response, the 1E6 TCR was also injected over a control sample (HLA-A*0201–ILAKFLHWL) that was deducted from the experimental data. (**A**) 1E6-A2-**MV**WGPDP**LYV** (approximate value); (**B**) 1E6-A2-**YLG**GPD**FPTI** (approximate value); (**C**) 1E6-A2-ALWGPDPAAA; (**D**) 1E6-A2-A**Q**WGPDPAAA; (**E**) 1E6-A2-**RQF**GPD**WIV**A; (**F**) 1E6-A2-**RQ**WGPDPAA**V**; (**G**) 1E6-A2-**YQF**GPD**FPTA**; and (**H**) 1E6-A2-**RQF**GPD**FPTI**. (**I**) ΔG values, calculated from SPR experiments, plotted against 1/EC_50_ (the reciprocal peptide concentration required to reach half-maximal 1E6 T cell killing) showing Pearson’s coefficient analysis (*r*) and *P* value (including approximate values from **A** and **B**). (**J**) Effective 2D affinity (*A_c_K_a_*) calculated using adhesion frequency assays, using at least 5 cell pairs, and calculated as an average of 100 cell cell contacts. (**K**) Effective 2D affinity plotted against 1/EC_50_ showing Pearson’s coefficient analysis (*r*) and *P* value.

We, and others, have previously demonstrated that antipathogenic TCRs tend to bind with stronger affinity compared with self-reactive TCRs (28), likely a consequence of the deletion of T cells with high-affinity self-reactive TCR during thymic selection. In accordance with this trend, the 1E6 TCR bound the natural preproinsulin peptide, ALWGPDPAAA, with the weakest affinity currently published for a human CD8^+^ T cell–derived TCR with a biologically relevant ligand (K_D_ > 200 μM; K_D_, equilibrium binding constant) (23). Surface plasmon resonance (SPR) analysis of the 1E6 TCR–pMHC interaction for all 7 APLs (Figure 2, A–H) demonstrated that stronger binding affinity (represented as ΔG°, kcal/mol) correlated well with the EC_50_ values (peptide concentration required to reach half-maximal 1E6 T cell killing) for each ligand, demonstrated by a Pearson’s correlation analysis value of 0.8 (*P* = 0.01) (Figure 2I). It should be noted that this correlation, although consistent with the T cell killing experiments, uses only approximate affinities calculated for the 2 weakest ligands. These experiments revealed 4 important findings. First, the 1E6 T cell could still functionally respond to peptide when the TCR binding affinity was extremely weak, e.g., the 1E6 TCR binding affinity for the A2-**MV**WGPDP**LYV** peptide was K_D_ = ~600 μM. Second, the 1E6 TCR bound to A2-**RQF**GPD**FPTI** with K_D_ = 0.5 μM, equivalent to the binding affinity of the very strongest antipathogen TCRs (29). Third, the 1E6 TCR bound to A2-**RQF**GPD**WIV**A peptide, within the *C. asparagiforme* proteome, with approximately 4-fold stronger affinity than A2-ALWGPDPAAA, demonstrating the potential for a pathogen-derived antigen to initiate a response to the self-derived sequence. Finally, these data demonstrate the largest range of binding affinities reported for a natural, endogenous human TCR of more than 3 logs of magnitude (A2-**MV**WGPDP**LYV** vs. A2-**RQF**GPD**FPTI**). To confirm the affinity spread detected by SPR, and to evaluate whether experiments performed using soluble molecules were biologically relevant to events at the T cell surface, we determined the effective 2D affinity of each APL using an adhesion frequency assay in which a human rbc coated in pMHC acted as an adhesion sensor (30, 31). In agreement with SPR experiments, the range of 2D affinities we detected differed by around 3 logs, with the A2-**MV**WGPDP**LYV** generating the weakest 2D affinity (2.6 × 10^–5^ *A*_c_*K*_a_ μm^4^) and A2-**RQF**GPD**FPTI** the strongest (4.5 × 10^–2^ *A*_c_*K*_a_ μm^4^) (Figure 2J). As with the 3D affinity measurements, the 2D affinity measurements correlated well with the EC_50_ values for each ligand (Figure 2K) demonstrating a strong correlation (Pearson’s correlation = 0.8, *P* = 0.01) between T cell antigen sensitivity and TCR binding affinity. Of note, these data demonstrate a close agreement between the 3D affinity values generated using SPR and 2D affinity values generated using adhesion frequency assays.

### The 1E6 TCR uses a consensus binding mode to engage multiple APLs.

**Figure 3:**
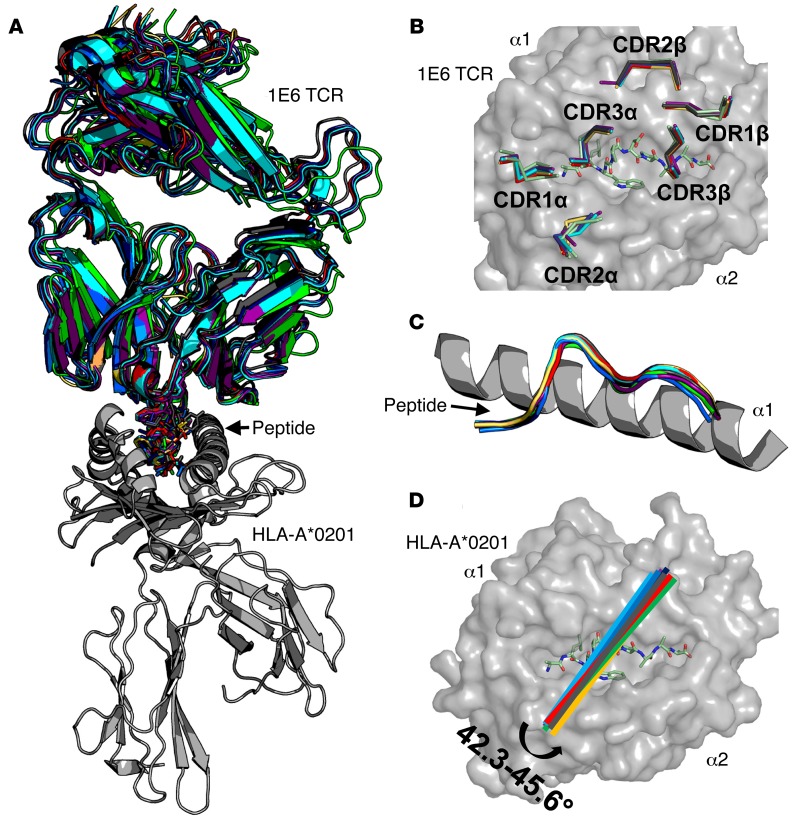
The 1E6 TCR uses a conserved binding mode to engage A2-ALWGPDPAAA and the APLs. (**A**) Superposition of the 1E6 TCR (multicolored illustration) in complex with all 7 APLs (multicolored sticks) and the A2-ALWGPDPAAA ligand (21) using the HLA-A*0201 (gray illustration) molecule to align all of the structures. The 1E6 TCR and each peptide are colored according to the APL used in the complex as in Figure 1. (**B**) Position of the 1E6 TCR CDR loops (multicolored lines) in each complex. The ALWGPDPAAA peptide (green sticks) is shown in the HLA-A*0201 binding groove (gray surface). (**C**) The Cα backbone conformation of each APL (multicolored illustration) in the context of the HLA-A*0201 α1 helices (gray illustration). (**D**) Crossing angle of the 1E6 TCR (multicolored lines) calculated using previously published parameters (51) in the context of the ALWGPDPAAA peptide (green sticks) bound in the HLA-A*0201 binding groove (gray surface).

Our previous structure of the 1E6-A2-ALWGPDPAAA complex demonstrated a limited binding footprint between the TCR and pMHC (23). The low number of contacts between the 2 molecules most likely contributed to the weak binding affinity of the interaction. In order to examine the mechanism by which the 1E6 TCR engaged a wide range of peptides with divergent binding affinities, we solved the structure of the 1E6 TCR in complex with all 7 APLs used in Figure 2. All structures were solved in space group P1 to 2–3 Å resolution with crystallographic R_work_/R_free_ ratios within accepted limits as shown in the theoretically expected distribution (ref. 32 and Supplemental Table 1). The 1E6 TCR used a very similar overall binding modality to engage all of the APLs, with root mean square deviation ranging between 0.81 and 1.12 Å^2^ (compared with 1E6-A2-ALWGPDPAAA). The relatively broad range of buried surface areas (1,670–1,920 Å^2^) did not correlate well with TCR binding affinity (Pearson’s correlation = 0.45, *P* = 0.2). The surface complementarity values (0.52–0.7) correlated slightly with affinity (Pearson’s correlation = 0.7, *P* = 0.05) but could not explain all differences in binding (Figure 3A and Table 2). The TCR CDR loops were in a very similar position in all complexes, apart from some slight deviations in the TCR β-chain (Figure 3B); the peptides were all presented in a similar conformation (Figure 3C); and there was minimal variation in crossing angles of the TCR (42.3°–45.6°) (Figure 3D). Overall, the 1E6 TCR used a canonical binding mode to engage each APL with the TCR α-chain positioned over the MHC class I (MHCI) α2-helix and the TCR β-chain over the MHCI α-1 helix, straddling the peptide cargo (29). However, subtle differences in the respective interfaces were apparent (discussed below) and resulted in altered binding affinities of the respective complexes.

### Interactions between the 1E6 TCR and different APLs are focused around a conserved GPD peptide motif.

**Figure 4:**
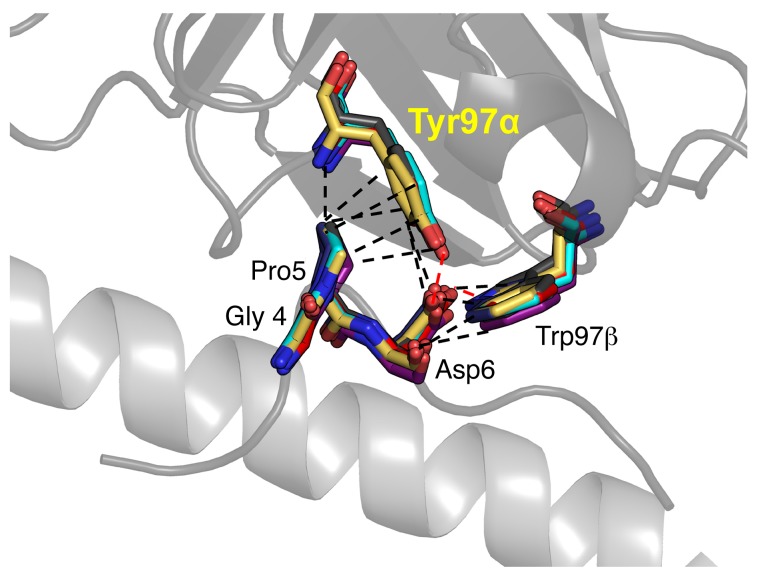
A conserved interaction with a GPD motif underpins the 1E6 TCR interaction with the APLs. Interaction between 1E6 TCR (gray illustration) residues Tyr97α and Tyr97β (the position of these side chains in the TCR in complex with all 7 APLs, and the previously reported A2-ALWGPDPAAA epitope, is shown in multicolored sticks; ref. 21) and the GPD peptide motif (the position of these side chains in all 7 APLs and A2-ALWGPDPAAA in complex with the 1E6 TCR is shown in multicolored sticks). The rest of the peptide, and the MHCα1 helix, are shown as a gray illustration.

We next performed an in-depth atomic analysis of the contacts between the 1E6 TCR and each APL to determine the structural basis for the altered T cell peptide sensitivities and TCR binding affinities (Table 2). Concomitant with our global analysis of 1E6 TCR binding to the APLs, we observed a common interaction element, consistent with our previous findings (23), that utilized TCR residues Tyr97α and Trp97β, forming an aromatic cap over a central GPD motif that was present in all of the APLs (Figure 4). Interactions between these 2 TCR and 3 peptide residues accounted for 41%–50% of the total contacts across all complexes (Table 2), demonstrating the conserved peptide centric binding mode utilized by the 1E6 TCR. This fixed anchoring between the 2 molecules was important for stabilization of the TCR-pMHC complex, as — although other peptides without the ‘GDP’ motif were tested and shown to activate the 1E6 T cell clone (3) — we were unable to measure robust affinities using SPR (data not shown). These data support the requirement for a conserved interaction between the 1E6 TCR and the GPD motif, as we observed in our previously published 1E6-A2-ALWGPDPAAA structure (23).

### Focused hotspot binding around a conserved GPD motif enables the 1E6 TCR to tolerate peptide degeneracy.

**Figure 5:**
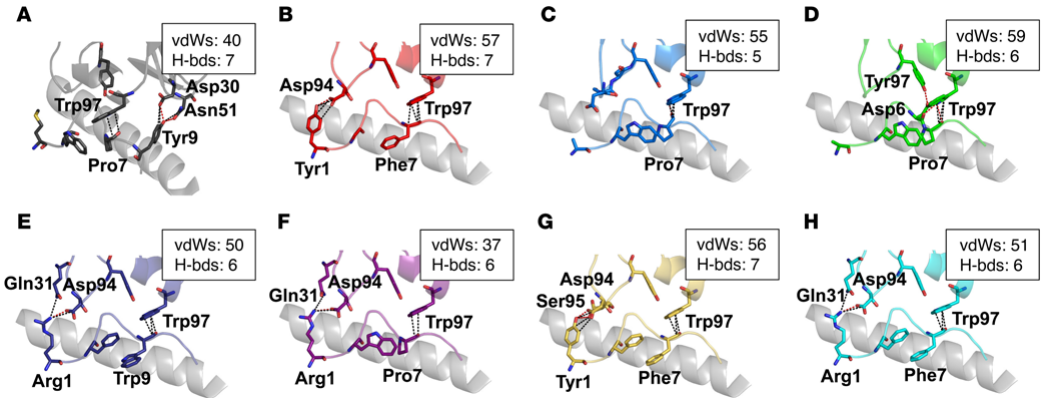
The 1E6 TCR makes distinct peptide contacts with peripheral APL residues. Interactions between the 1E6 TCR and peptide residues outside of the conserved GPD motif. The MHCα1 helix is shown in gray illustrations. Hydrogen bonds are shown as red dotted lines; van der Waals (vdW) contacts are shown as black dotted lines. Boxes show total contacts between the 1E6 TCR and each peptide ligand. (**A**) Interaction between the 1E6 TCR (black illustration and sticks) and A2-**MV**WGPD**PLYV** (black illustration and sticks). (**B**) Interaction between the 1E6 TCR (red illustration and sticks) and A2-**YLG**GPDFPTI (red illustration and sticks). (**C**) Interaction between the 1E6 TCR (blue illustration and sticks) and A2-ALWGPDPAAA (blue illustration and sticks) reproduced from previous published data (21). (**D**) Interaction between the 1E6 TCR (green illustration and sticks) and A2-A**Q**WGPDPAAA (green illustration and sticks). (**E**) Interaction between the 1E6 TCR (dark blue illustration and sticks) and A2-**RQF**GPD**WIV**A (dark blue illustration and sticks). (**F**) Interaction between the 1E6 TCR (purple illustration and sticks) and A2-**RQW**GPDPAA**V** (purple illustration and sticks). (**G**) Interaction between the 1E6 TCR (yellow illustration and sticks) and A2-**YQF**GPD**FPTA** (yellow illustration and sticks). (**H**) Interaction between the 1E6 TCR (cyan illustration and sticks) and A2-**RQF**GPD**FPTI** (cyan illustration and sticks).

Although the 1E6 TCR formed a similar overall interaction with each APL, the stabilization between the TCR and the GPD motif enabled fine differences in the contact network with both the peptide and MHC surface that allowed discrimination between each ligand (Figure 5). For example, the 1E6 TCR made only 47 peptide contacts with A2-**MV**WGPDP**LYV** (K_D_ = ~600 μM) compared with 63 and 57 contacts with A2-**YQF**GPD**FPI**A (K_D_ = 7.4 μM) and A2-**RQF**GPD**FPTI** (K_D_ = 0.5 μM), respectively. Although the number of peptide contacts was a good predictor of TCR binding affinity for some of the APLs, for others, the correlation was poor (Pearson’s correlation = 0.045, *P* = 0.92), possibly because of different resolutions for each complex structure. For example, the 1E6 TCR made 64 peptide contacts with A2-**YLG**GPD**FPTI** (K_D_ = ~400 μM) compared with 43 contacts with A2-R**Q**WGPDPAAV (K_D_ = 7.8 μM).

**Figure 6:**
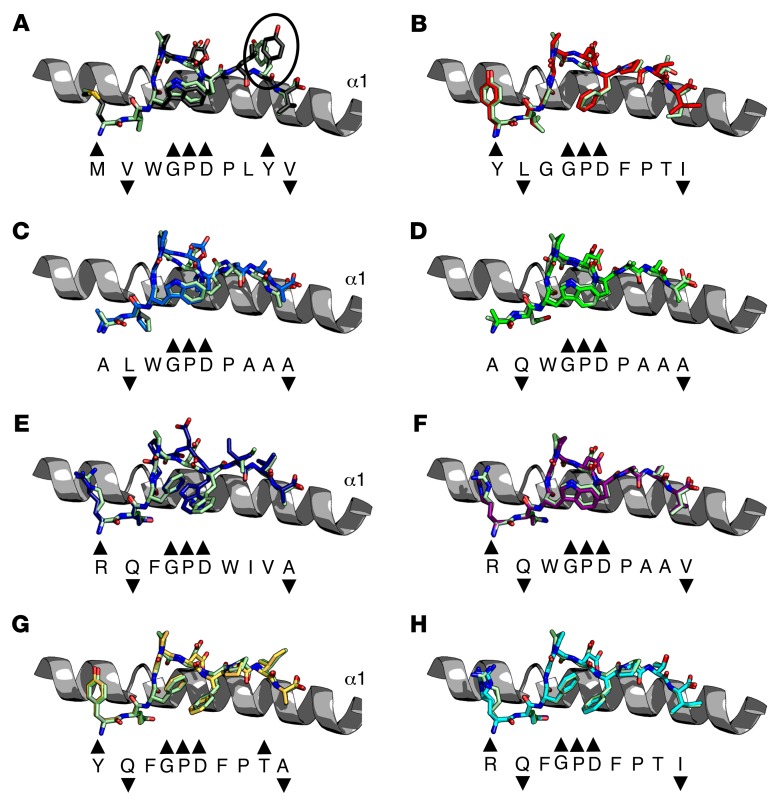
Comparison of ligated and unligated APLs. Superposition of each APL in unligated form and ligated to the 1E6 TCR. All unligated pMHCs are shown as light green illustrations. Peptide sequences are shown underneath each structure aligned with the peptide structure. Black arrows denote the direction of the side chain. Upward arrows indicates solvent exposed, downward arrows indicates anchor position, and no arrow indicates an intermediate position. (**A**) A2-**MV**WGPD**PLYV** (black sticks). A large conformational shift was observed for Tyr8 in the ligated versus unligated states (black circle). (**B**) A2-**YLG**GPD**FPTI** (red sticks). (**C**) A2-ALWGPDPAAA (blue sticks) reproduced from previous published data (21). (**D**) A2-A**Q**WGPDPAAA (green sticks). (**E**) A2-**RQF**GPD**WIV**A (dark blue sticks). (**F**) A2-**RQ**WGPDPAA**V** (purple sticks). (**G**) A2-**YQF**GPD**FPTA** (yellow sticks). (**H**) A2-**RQF**GPD**FPTI** (cyan sticks).

The most important peptide modification in terms of generating new contacts was peptide position 1. The stronger ligands all encoded larger side chains (Arg or Tyr) at peptide position 1 (Figure 5, E–H), enabling interactions with 1E6 that were not present in the weaker APLs that lacked large side chains in this position (Figure 5, A, C, and D). We have previously shown that the 1E6 TCR uses a rigid lock-and-key mechanism during binding to A2-ALWGPDPAAA (23). These data demonstrated that the unligated structure of the 1E6 TCR was virtually identical to its ligated counterparts. In order to determine whether any of the APLs required an induced fit mechanism during binding that could explain the difference in free binding energy (ΔG) between each complex (Table 2), we solved the unligated structures of all 7 APLs (the A2-ALWGPDPAAA structure has been previously published and was used in this comparison, ref. 23) (Figure 6 and Supplemental Table 2). The unligated A2-**MV**WGPDP**LYV** (K_D_ = ~600 μM) structure revealed that the side chain Tyr9 swung around 8 Å in the complex structure, subsequently making contacts with TCR residues Asp30β and Asn51β (Figure 6A and Figure 5A, respectively). This movement could result in an entropic penalty contributing to the weak TCR binding affinity we observed for this ligand. Additional small movements in the Cα backbone of the peptide around peptide residue Asp6 were apparent in the A2-**YLG**GPD**FPTI** (K_D_ = ~400 μM), A2-ALWGPDPAAA (K_D_ = ~208 μM), and A2-**RQF**GPD**WIV**A (K_D_ = 44.4 μM) structures (Figure 6, B, C, and E). The unligated structures of A2-A**Q**WGPDAAA, A2-**RQ**WGPDPAA**V**, A2-**YQF**GPD**FPI**A, and A2-**RQF**GPD**FPTI** were virtually identical when in complex with 1E6 (Figure 6, D and F–H). Apart from the case of A2-A**Q**WGPDAAA (K_D_ = 61.9 μM), these observations support the conclusion that the higher-affinity ligands required less conformational melding during binding, which could be energetically beneficial (lower entopic cost) during ligation with the 1E6 TCR.

### Peptide modifications alter the interaction between the 1E6 TCR and the MHC surface.

**Figure 7:**
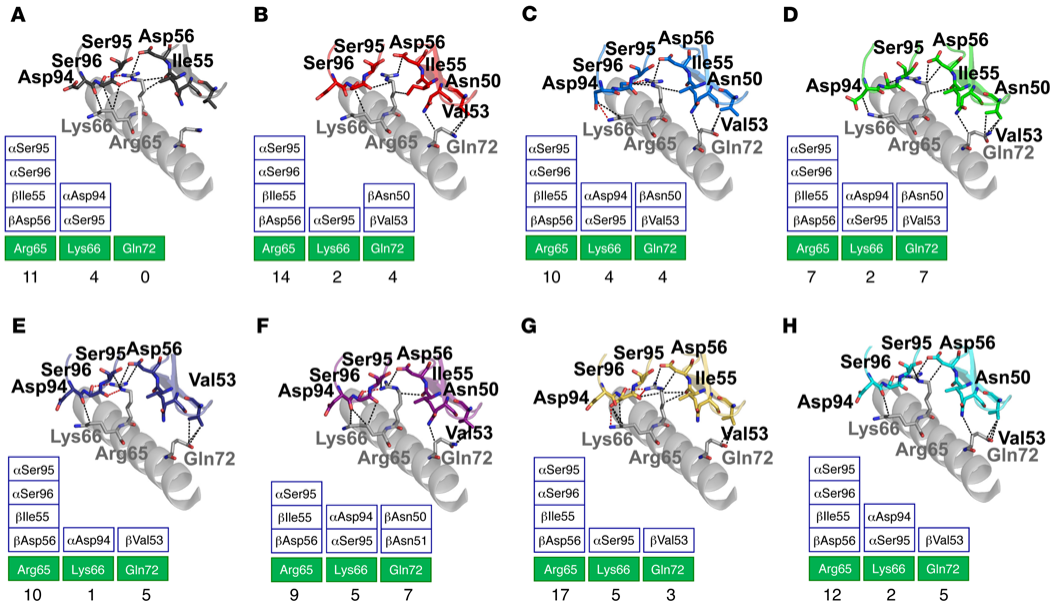
The 1E6 TCR makes distinct peptide contacts with the MHC surface depending on the peptide cargo. Interactions between the 1E6 TCR and the MHC α1 helix residues Arg65, Lys66, and Gln72. Hydrogen bonds are shown as red dotted lines; vdW contacts are shown as black dotted lines. MHCα1 helix are shown in gray illustrations. Boxes show total contacts between the 1E6 TCR and these key residues (green boxes are MHC residues; white boxes are TCR residues). (**A**) Interaction between the 1E6 TCR (black illustration and sticks) and A2-**MV**WGPD**PLYV** (black illustration and sticks). (**B**) Interaction between the 1E6 TCR (red illustration and sticks) and A2-**YLG**GPD**FPTI** (red illustration and sticks). (**C**) Interaction between the 1E6 TCR (blue illustration and sticks) and A2-ALWGPDPAAA (blue illustration and sticks) reproduced from previous published data (21). (**D**) Interaction between the 1E6 TCR (green illustration and sticks) and A2-A**Q**WGPDPAAA (green illustration and sticks). (**E**) Interaction between the 1E6 TCR (dark blue illustration and sticks) and A2-**RQF**GPD**WIVA** (dark blue illustration and sticks). (**F**) Interaction between the 1E6 TCR (purple illustration and sticks) and A2-**RQ**WGPDPAA**V** (purple illustration and sticks). (**G**) Interaction between the 1E6 TCR (yellow illustration and sticks) and A2-**YQF**GPD**FPT**A (yellow illustration and sticks). (**H**) Interaction between the 1E6 TCR (cyan illustration and sticks) and A2-**RQF**GPD**FPTI** (cyan illustration and sticks).

In addition to changes between the TCR and peptide component, we also observed that different APLs had different knock-on effects between the TCR and MHC. MHC residue Arg65 that forms part of the MHC restriction triad (Arg65, Ala69, and Gln155) (15, 33) played a central role in TCR-MHC contacts, with Gln155 playing a less important role and Ala69 playing no role in binding at the interface (Figure 7). Generally, the weaker-affinity APLs made fewer contacts with the MHC surface (27–29 interactions) compared with the stronger-affinity APLs (29–35 contacts), consistent with a better Pearson’s correlation value (0.55) compared with TCR-peptide interactions versus affinity (0.045). For instance, contacts were made between TCR residue Val53β and MHC residue Gln72 in all APLs except for in the weakest affinity ligand pair, 1E6-A2-**MV**WGPDP**LYV**, in which a subtle change in TCR conformation — probably mediated by different peptide contacts — abrogated this interaction (Figure 7A).

### An energetic switch from unfavorable to favorable entropy (order-to-disorder) correlates with antigen potency.

**Figure 8:**
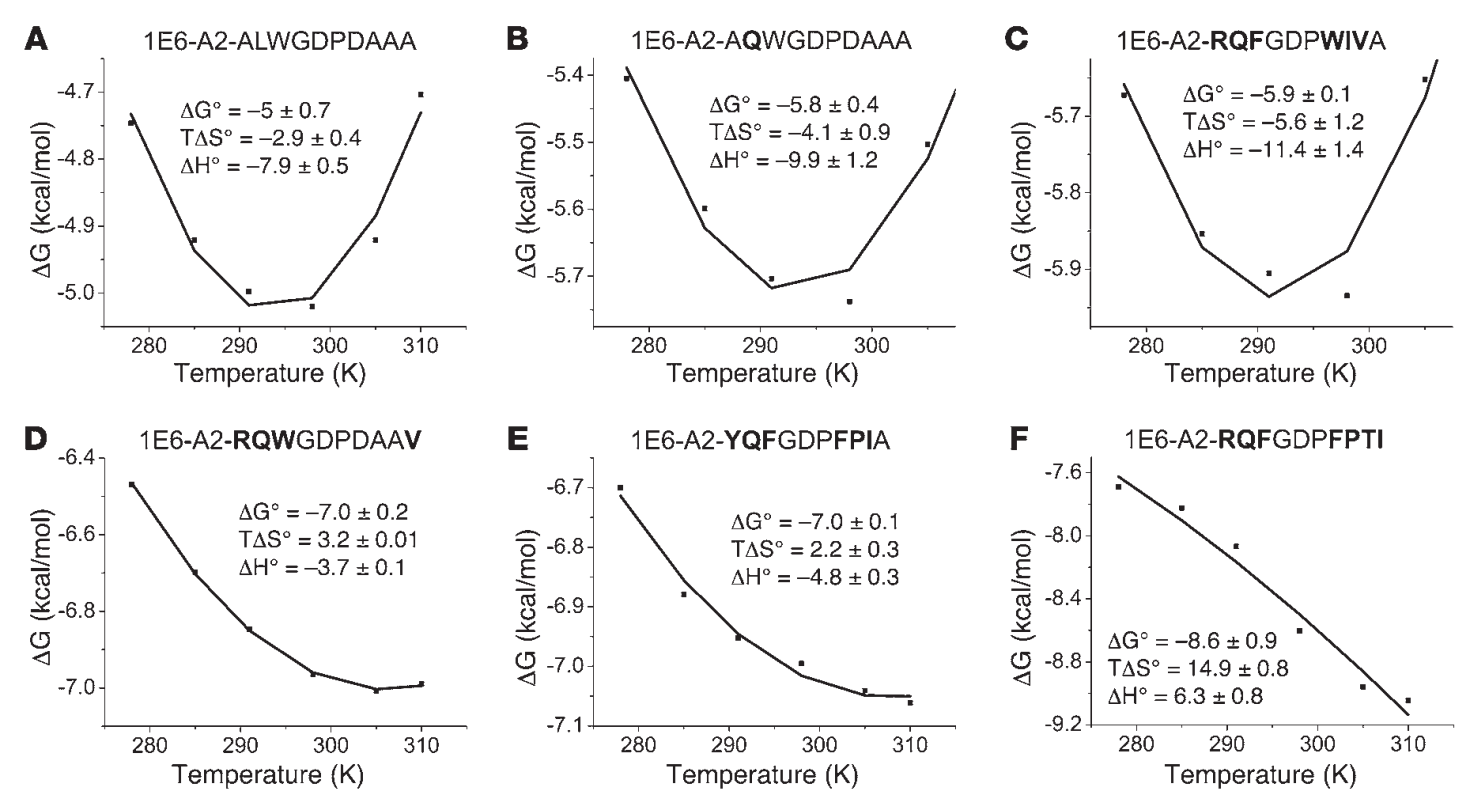
Thermodynamic analysis of the 1E6 TCR with A2-ALWGPDPAAA and the APLs. Eight serial dilutions of the 1E6 TCR were injected, in duplicate, over each immobilized APL and A2-ALW at 5°C, 13°C, 18°C, 25°C, 30°C, and 37°C. The equilibrium binding constant (K_D_) values were calculated using a nonlinear curve fit (*y* = [P_1_*x*]/[P_2_ + X]), and thermodynamic parameters were calculated according to the Gibbs-Helmholtz equation (ΔG° = ΔH − TΔS°). The binding free energies, ΔG° (ΔG° = RTlnK_D_), were plotted against temperature (K) using nonlinear regression to fit the 3-parameters van’t Hoff equation (RT ln K_D_ = ΔH° – TΔS° + ΔCp°[T-T_0_] – TΔCp° ln [T/T_0_] with T_0_ = 298 K). (**A**) 1E6-A2-ALWGPDPAAA; (**B**) 1E6-A2-A**Q**WGPDPAAA; (**C**) 1E6-A2-**RQF**GPD**WIV**A; (**D**) 1E6-A2-**RQW**GPDPAA**V**, (**E**) 1E6-A2-**YQF**GPD**FPT**A; and (**F**) 1E6-A2-**RQF**GPD**FPTI**.

Our analysis of the contact network provided some clues that could explain the different antigen potencies and binding affinities between the 1E6 TCR and the different APLs. However, there were clear outliers in which the number of contacts did not match with the strength/potency of the interaction. For example, the 1E6 TCR bound to A2-**RQ**WGPDPAA**V** with the third strongest affinity (K_D_ = 7.8 μM) but made fewer contacts than with A2-ALWGPDPAAA (K_D_ = ~208 μM) (Table 2). However, it is not necessarily the quantity of contacts that determines the strength of an interaction, but the quality of the contacts. Thus, we performed an in-depth thermodynamic analysis of 6 of the ligands under investigation (Figure 8 and Supplemental Table 3). The weak binding affinity between 1E6 and A2-**MV**WGPDP**LYV** and A2-**YLG**GPD**FPTI** generated thermodynamic data that were not robust enough to gain insight into the enthalpic (ΔH°) and entropic (TΔS°) changes that contributed to the different binding affinities/potencies for each APL. The overall free binding energies (ΔG°) were between –4.4 and –8.6 kcal/mol, reflecting the wide range of TCR binding affinities we observed for the different APLs. The enthalpic contribution in each complex did not follow a clear trend with affinity, with all but the 1E6-A2-**RQF**GPD**FPTI** interaction (ΔH° = 6.3 kcal/mol) generating an energetically favorable enthalpy value (ΔH° = –3.7 to –11.4 kcal/mol); this indicated a net gain in electrostatic interactions during complex formation. However, there was a clear switch in entropy between the weaker-affinity and stronger-affinity ligands, indicated by a strong Pearson’s correlation value between entropy and affinity (Pearson’s correlation value 0.93, *P* =0.007). For instance, the A2-ALWGPDPAAA, A2-A**Q**WGPDAAA, and A2-**RQF**GPD**WIV**A (K_D_ = ~208 μM, K_D_ = 61.9 μM, and K_D_ = 44.4 μM, respectively) were all entropically unfavorable (TΔS° = –2.9 to –5.6 kcal/mol), indicating a net change from disorder to order. Conversely, the stronger-affinity ligands A2-**RQ**WGPDPAA**V** (K_D_ = 7.8 μM), A2-**YQF**GPD**FPIA** (K_D_ = 7.4 μM), and A2-**RQF**GPD**FPTI** (K_D_ = 0.5 μM) exhibited favorable entropy (TΔS° = 2.2 to 14.9 kcal/mol), indicating an order-to-disorder change during binding, possibly through the expulsion of ordered water molecules. Furthermore, the structures of the unligated pMHCs demonstrated that, for these stronger-affinity ligands, there was less conformational difference between the TCR ligated pMHCs compared with the weaker-affinity ligands (Figure 6). The potential requirement for a larger degree of induced fit during binding to these weaker-affinity ligands is consistent with the larger entropic penalties observed for these interactions.

### Potential epitopes for 1E6 TCR occur commonly in the viral proteome.

We searched a database of over 1,924,572 unique decamer peptides from the proteome of viral pathogens that are known, or strongly suspected, to infect humans. Three hundred forty-two of these decamers conformed to the motif xxxGPDxxxx. Of these, 53 peptides contained the motif xOxGPDxxxO, where O is one of the hydrophobic amino acid residues A,V, I, L, M, Y, F, and W that might allow binding to HLA-A*0201 (Supplemental Table 4). Thus, there are many pathogen-encoded peptides that could act as agonists for the 1E6 T cell beyond the **MV**WGPDP**LYV** and **RQF**GPD**WIV**A sequences studied here. Extension of these analyses to include the larger genomes of bacterial pathogens would be expected to considerably increase these numbers. The binding affinity of the 1E6 TCR interaction with A2-**RQF**GPD**WIV**A is considerably higher than with the disease-implicated A2-ALWGPDPAAA sequence (K_D_ = 44.4 μM and K_D_ > 200 μM, respectively), highlighting how a pathogen-derived sequence might be capable of priming a 1E6-like T cell.

## Discussion

T cell antigen discrimination is governed by an interaction between the clonally expressed TCR and pMHC, mediated by the chemical characteristics of the interacting molecules. It has recently become clear that TCR cross-reactivity with large numbers of different pMHC ligands is essential to plug holes in T cell immune coverage that pathogens could exploit (5). Flexibility at the interface between the TCR and pMHC, demonstrated in various studies (29), has been suggested as a mechanism mediating T cell cross-reactivity with multiple distinct epitopes. This notion is attractive because the CDR loops, which form the TCR antigen-binding site, are usually the most flexible part of the TCR and have the ability to mold around differently shaped ligands. Focused binding around a minimal peptide motif has also been implicated as an alternative mechanism enabling TCR cross-reactivity (1, 11–14, 16, 22, 34). Notably among these studies, Garcia and colleagues recently used the alloreactive murine TCR-MHC pair of the 42F3 TCR and H2-L^d^ to demonstrate recognition of a large number of different peptides via conserved hotspot contacts with prominent up-facing peptide residues (22).

Sethi and colleagues recently demonstrated that the MHCII-restricted Hy.1B11 TCR, which was isolated from a patient with multiple sclerosis, could anchor into a deep pocket formed from peptide residues 2, 3, and 5 (from MBP_85–99_ bound to HLA-DQ1) (19). This motif was conserved in at least 2 potential foreign peptides, originating from *Herpes simplex* virus and *Pseudomonas aeruginosa*, enabling TCR recognition of foreign epitopes (19). Although these data provided some clues into the molecular mechanism of T cell recognition, there still remain several gaps in our understanding. First, we currently know nothing about how human MHCI–restricted TCRs mediate cross-reactivity in the context of a clinically relevant model of autoimmunity, thought to be a major pathway of disease initiation in several autoimmune diseases (35). Second, molecular studies have not yet revealed a broad set of rules that determine TCR cross-reactivity because, with the exception of the allo–TCR-MHC pair of the 42F3 TCR and H2-L^d^ that did not encounter each other during T cell development (22), studies have been limited to structures of a TCR with only 2 or 3 different ligands (10, 14, 21, 36–40). Finally, no studies have included characterization of pathogen-derived ligands recognized by self-reactive T cells with greater potency than the autoantigen, a potentially important facet to break self-tolerance.

Here, we investigated a highly cross-reactive MHCI-restricted TCR isolated from a patient with T1D that recognizes an HLA-A*0201–restricted preproinsulin signal peptide (ALWGPDPAAA_15–24_) (3, 23, 25). Human CD8^+^ T cell clones expressing TCRs with this specificity mediate the destruction of β cells, have been found in islets early in infection, and are proposed to be a major driver of disease (8, 26). We solved the structure of the 1E6 TCR with 7 APLs to enable a comprehensive analysis of the molecular basis of TCR degeneracy. The epitopes we selected exhibited a broad range of potencies and could activate the 1E6 T cell clone at exogenously supplied concentrations more than 4 logs apart. Overall, the difference in antigen potency correlated well with the binding energy (ΔG° kcal/mol) of the 1E6 TCR for the different epitopes, which ranged from values of ΔG° = ~–4.4 to –8.6 kcal/mol (calculated from 3D affinity data) or 2D affinity values of *A*_c_*K*_a_ = 2.5 × 10^–5^ to 4.4 × 10^–2^ μm^4^. The weaker end of this spectrum extends our understanding of the limits in which T cells can functionally operate in terms of TCR 3D binding affinity and is in line with the types of very low affinity, yet fully functional self-reactive CD8^+^ T cells we have observed in tumor-infiltrating lymphocytes (41–43). Previous studies of autoreactive TCRs have shown that their binding mode is generally atypical, either due to an unusual binding manner (19, 44–47), weak TCR binding affinity (23, 36), an unstable pMHC (48, 49), or a combination of these factors. Our data demonstrate the potential for an autoreactive TCR to bind with a conventional binding mode to a stable pMHC with antipathogen-like affinity (K_D_ = 0.5 μM) depending on the peptide sequence. Our structural analysis revealed that the 1E6 TCR bound with a conserved conformation across all APLs investigated. This binding orientation was mediated through a focused interaction with TCR residues Tyr97α and Trp97β that formed an aromatic cap over a central ‘GDP’ motif that was common to all APLs. We have previously demonstrated the importance of the GPD motif using a peptide library scan (23), as well as a CPL scan approach (3). Although the 1E6 T cell was able to activate weakly with peptides that lacked this motif, we were unable to robustly measure binding affinities or generate complex structures with these ligands, highlighting the central role of this interaction during 1E6 T cell antigen recognition. This hotspot binding, defined as a localized cluster of interactions that dominate binding energy during protein-protein interactions (50), has been previously shown to contribute to TCR recognition of MHC as a mechanism that tunes T cell cross-reactivity by providing fixed anchor points that enable TCRs to tolerate a variable peptide cargo (1, 51–53). Alternatively, interactions between the TCR and peptide have been shown to dominate the energetic landscape during ligand engagement, ensuring that T cells retain peptide specificity (54, 55). The binding mechanism utilized by the 1E6 TCR during pMHC recognition is consistent with both of these models. Ligand engagement is dominated by peptide interactions, but hotspot-like interactions with the central GPD motif enable the 1E6 TCR to tolerate peptide residues that vary outside of this region, explaining how T cells expressing this TCR may cross-react with a large number of different peptides (3). These findings are also analogous to the observed binding mode of the Hy.1B11 TCR, in which one aromatic residue of the TCR CDR3α loop anchored into a pocket created by a conserved peptide motif (19). In both of these examples, self-recognition is mediated by TCR residues with aromatic side chains. These large, generally hydrophobic amino acids can form strong interactions with other residues through π-π stacking. Combined with evidence demonstrating that aromatic side chains are conserved in the CDR2 loops of TCRs from many species (56), we speculate that these aromatic residues could impart a level of “stickiness” to TCRs, which might be enriched in an autoimmune setting when the TCR often binds in a nonoptimal fashion.

Despite some weak statistical correlation between the surface complementarity (SC) and affinity, closer inspection of the interface revealed no obvious structural signature that could definitively explain the differences in antigen potency and TCR binding strength between the different ligands. However, similar to our findings in other systems (57–59), modifications to residues outside of the canonical central peptide bulge were important for generating new interactions. For example, all of the stronger ligands encoded larger side chains (Arg or Tyr) at peptide position 1 that enabled new interactions with 1E6 not present with the Ala at this position in the natural preproinsulin peptide. These data also explain our previous findings that alteration of the anchor residue at peptide position 2 (Leu-Gln) has a direct effect on 1E6 TCR binding affinity (60) because our structural analysis demonstrated that 1E6 made 3 additional bonds with A2-A**Q**WGPDPAAA compared with A2-ALWGPDPAAA, consistent with the >3-fold stronger binding affinity. We have recently demonstrated how a suboptimal position 2 anchor in a melanoma-derived antigen can improve TCR binding through a similar mechanism (58). These results challenge the notion that the most potent peptide antigens exhibit the greatest pMHC stability and have implications for the design of anchor residue–modified heteroclitic peptides for vaccination.

Early thermodynamic analysis of TCR-pMHC interactions suggested a common energetic signature, driven by favorable enthalpy (generally mediated through an increase in electrostatic interactions) and unfavorable entropy (changes from disorder to order) (61, 62). These parameters aligned well with structural data, demonstrating that TCRs engaged pMHC using an induced fit binding mode (63). However, more recent data have shown that TCRs can utilize a range of energetic strategies during pMHC binding, currently with no obvious pattern in terms of TCR affinity, binding mechanism, or specificity (pathogen, cancer, or self-ligands) (12, 57, 64, 65). Although no energetic signature appears to exist for different TCRs, we used thermodynamic analysis here to explore whether changes in energetics could help explain ligand discrimination by a single TCR. This analysis demonstrated a strong relationship (according to the Pearson’s correlation analysis) between the energetic signature used by the 1E6 TCR and the sensitivity of the 1E6 T cell clone to different APLs. The weaker APL ligands were characterized by favorable enthalpy and unfavorable entropy, whereas the stronger ligands progressively shifted to favorable entropy. These differences were consistent with a greater degree of movement between the unligated and ligated pMHCs for the weaker ligands, suggesting a greater requirement for disorder-to-order changes during TCR binding (54, 66, 67). Thus, the enhanced antigen potency was probably mediated through a shift from an induced fit to a lock-and-key interaction between the stronger ligands (less requirement for energetically unfavorable disorder-to-order changes), resulting in a more energetically favorable ΔG value.

Importantly, the preproinsulin-derived epitope was one of the least potent peptides, demonstrating that the 1E6 T cell clone had the ability to respond to different peptide sequences with far greater potency. The **RQF**GPD**WIV**A peptide, which was substantially more potent than the preproinsulin peptide, is within the proteome of a common human pathogen (*C. asparagiforme*), demonstrating the potential for an encounter between a naive 1E6-like T cell and a foreign peptide with a more potent ligand that might then break self-tolerance. Indeed, we found over 50 decamer peptides from the proteome of likely, or known, human viral pathogens alone that contained both the conserved central GPD motif and anchor residues at positions 2 and 10 that would enable binding to HLA-A*02:01. Further experiments will be required to determine whether any naturally presented, human pathogen–derived peptides act as active ligands for 1E6, but our work presented here demonstrates that it is at least feasible for an autoimmune TCR to bind to a different peptide sequence that could be present in a pathogen proteome with substantially higher affinity and potency than the interaction it might use to attack self-tissue.

In summary, this investigation into the molecular basis of T cell cross-reactivity using a clinically relevant cytotoxic CD8^+^ T cell clone that kills human pancreatic β cells (24, 25) provides answers to a number of previously outstanding questions. First, our data shows that a single TCR has the potential to functionally (assessed through T cell activation) bind to different ligands with affinities ranging across 3 orders of magnitude. Second, this is the first example in which ligands have been identified and characterized for a human autoreactive TCR that are substantially more potent than the natural self-ligand, demonstrating the potential for a pathogenic ligand to break self-tolerance and prime self-reactive T cells. Third, this first structural analysis of a cross-reactive human MHCI–restricted autoimmune TCR showed that degeneracy was mediated through TCR-pMHC anchoring by a conserved minimal binding peptide motif. Finally, TCR ligand discrimination was characterized by an energetic shift from an enthalpically to entropically driven interaction. Our demonstration of the molecular mechanism governing cross-reactivity by this preproinsulin reactive human CD8^+^ T cell clone supports the notion first put forward by Wucherpfennig and Strominger that molecular mimicry could mediate autoimmunity (7–9) and has far-reaching implications for the complex nature of T cell antigen discrimination.

## Methods

### T cell maintenance and culture.

The 1E6 T cell clone was generated as previously described (25) and stored in vapor phase liquid nitrogen in freezing buffer (90% FCS and 10% DMSO). Cells were defrosted rapidly in a 37°C water bath until a small amount of frozen cells were left and then immediately washed in 15–20 ml of R10 media (RPMI 1640 with 10% FCS, 100 IU/ml penicillin, 100 μg/ml streptomycin, 2 mM l-glutamine) by centrifuging at 300 *g* for 5 minutes. Defrosted cells were cultured in T cell media: R10 with 1× nonessential amino acids, 1 mM sodium pyruvate, 10 mM HEPES buffer (all from Invitrogen), 20 IU/ml of IL-2 (aldesleukin, brand name Proleukin, Prometheus) and 25 ng/ml IL-15 (PeproTech), for 24 hours; then, 0.75 × 10^6^ to 1.5 × 10^6^ cells expanded by coculture with 15 × 10^6^ irradiated (3,100 cGy) PBMCs from 3 donors in a 25 cm^2^ tissue culture flask with 1 μg/ml of phytoheamagglutinin (Alere Inc.) and T cell media as above. The clone was transferred to 24-well tissue culture plates (3 × 10^6^ to 4 × 10^6^ per well in 2 ml) at day 7, and the IL-2 increased to 200 IU/ml. For the purpose of this study, the clone was passaged 3 times and used between weeks 2 and 4 after expansion.

### T cell activation assays and tetramer staining.

The [^51^Cr] release cytotoxicity assay was performed as previously described (43). Target A2 CIR cells were labeled for 1 hour at 37°C with 30 μCi chromium (sodium chromate in normal saline, PerkinElmer) per 1 × 10^6^ cells, washed with R10, and allowed to leach for a further hour at 37°C in R10 to remove any excess chromium from the cells. After chromium labeling, target cells were washed and plated at 1,000 cells/well in 96-well tissue culture plates and pulsed with peptide at the indicated concentrations for 2 hours at 37°C. T cells were added to give the desired T cell/target cell (5:1) ratio and a final volume of 150 μl R10. Target cells were also incubated alone or with 1% Triton X-100 detergent (Sigma-Aldrich) to give the spontaneous and total chromium released from the target cells, respectively. After overnight incubation, at 37°C and 5% CO_2_, the supernatants were both (i) assayed for MIP1β by ELISA (R&D Systems) and (ii) harvested (10% of total volume), mixed with 150 μl Optipahse supermix scintillation mixture (PerkinElmer) in 96-well polyethylene terephthalate plates (PerkinElmer), and sealed; the amount of released chromium was measured indirectly on a 1450 Microbeta counter (PerkinElmer). The percentage of specific target cell lysis by T cells was calculated according to the following formula: (experimental release [with T cells and target cells] − spontaneous release from target cells)/(total release from target cells − spontaneous release from target cells) × 100. Experiments were independently completed in triplicate. Tetrameric pMHCI reagents (tetramers) were constructed by the addition of PE-conjugated streptavidin (Invitrogen) at a pMHCI/streptavidin molar ratio of 4:1. A total of 50,000 T cells were stained with PE-conjugated tetramer (25 μg/ml) folded around the indicated peptides for 30 minutes on ice and washed with PBS before staining with 2 μl (1:40 dilution of the DMSO stock in PBS) of the violet LIVE/DEAD fixable dead cell stain Vivid (Invitrogen) for 5 minutes at room temperature before direct addition of 2 μl of anti–CD8-APC antibody (clone BW135/80, Miltenyi Biotec) and incubated for a further 20 minutes on ice before being washed in FACS buffer (2% FCS in PBS). Data were acquired using a FACSCanto II flow cytometer (BD Biosciences) and analyzed with FlowJo software (Tree Star Inc.).

### Protein expression, refolding, and purification.

The 1E6 TCR, HLA-A*0201, and human β2m chain were generated as described previously (23). The 1E6 TCR and HLA-A*0201 peptide variants were refolded and purified as described previously (23). Biotinylated pMHCI and pMHC tetramer were prepared as previously described (68).

### pMHC stability assays.

Thermal stability of the HLA-A*0201–peptide complexes was assessed by circular dichroism (CD) spectroscopy monitoring the change in ellipticities at 218 nm. Data were collected, in duplicate, using a nitrogen-flushed Module B end-station spectrophotometer at the B23 Synchrotron Radiation CD Beamline at the Diamond Light Source (DLS) (69). Samples were prepared in phosphate buffered saline, pH 7.4, and concentrated to ~10 mM. Spectra were measured every 5°C over a temperature range between 5°C and 90°C with 5 minutes of equilibration time for each temperature. Four scans were acquired using an integration time of 1 second, a path length of 0.02 cm, and a slit width of 0.5 mm equivalent to a 1.2-nm bandwidth. Reversibility was monitored by measuring the spectrum at 20°C after cooling from 90°C with 30 minutes of incubation. Melting curves were analyzed assuming a 2-state trimer-to-monomer transition from the native (N) to unfolded (U) conformation N_3_ ↔ 3U with an equilibrium constant K = (U)^3^/(N_3_) = F/(3c^2^[1-F]^3^), where F and c are the degree of folding and protein concentration, respectively. Data were fitted as described (70). Fitted parameters were the melting temperature T_m_, van’t Hoff’s enthalpy ΔH_vH_, and the slope and intercept of the native baseline. As all protein complexes aggregated to various degrees upon unfolding, the ellipticity of the unfolded state was set as a constant of –4,500 deg cm^2^/dmol (71, 72).

### SPR analysis.

Binding analysis was performed using a BIAcore T200 equipped with a CM5 sensor chip as previously described (73). Binding analysis was performed 3× in independent experiments using pMHC monomers generated in-house. Approximately 200–500 RU of each HLA-A*0201–peptide complex was attached to the CM5 sensor chip at a slow flow rate of 10 μl/min to ensure uniform distribution on the chip surface. The 1E6 TCR was purified and concentrated to approximately 40–350 μM on the same day of SPR analysis to reduce the likelihood of TCR aggregation. For equilibrium analysis, 10 serial dilutions were prepared in triplicate for each sample and injected over the relevant sensor chips at 25°C. TCR was injected over the chip surface using kinetic injections at a flow rate of 45 μl/min using HLA-A*0201–E**L**AGIGILTV as a negative control surface on flow cell 1. For the thermodynamics experiments, this method was repeated at the following temperatures: 5°C, 13°C, 18°C, 25°C, 30°C, and 37°C. Results were analyzed using BIAevaluation 3.1, Excel, and Origin 6.0 software. The K_D_ values were calculated assuming a 1:1 interaction by plotting specific equilibrium-binding responses against protein concentrations, followed by nonlinear least squares fitting of the Langmuir binding equation. The thermodynamic parameters were calculated using the nonlinear van’t Hoff equation (RT ln K_D_ = ΔH° –TΔS° + ΔCp°[T-T_0_] – TΔCp° ln [T/T_0_]) with T_0_=298 K.

### Adhesion frequency assay.

We used an adhesion frequency assay to measure the 2D affinity of TCR-pMHC interactions at the cell membrane as previously described (30). Briefly, human 1E6 T cells were mounted onto 1 micropipette, and, on the other pipette, human rbcs coated with pMHC by biotin-streptavidin coupling served as both a surrogate APC and an adhesion sensor for detecting the TCR-pMHC interaction. Site densities of TCR and pMHC were measured by flow cytometry as previously described (74). All assays were performed using at least 5 cell pairs and calculated as an average of 100 cell-cell contacts.

### Crystal structure determination.

All protein crystals were grown at 18°C by vapor diffusion via the sitting drop technique. Each pMHCI (200 nl, 10 mg/ml) in crystallization buffer (10 mM TRIS [pH 8.1] and 10 mM NaCl) was added to 200 nl of reservoir solution. HLA-A*0201–**MV**WGPD**PLYV** (A2-MVW) crystals were grown in 0.2 M ammonium chloride, 0.1 M TRIS (pH 8), 20% PEG 6000; HLA-A*0201–**YLG**GPD**FPTI** (A2-YLG) crystals were grown in 0.2 M sodium nitrate, 0.1 M BIS TRIS propane (pH 6.5), 20% PEG 3350; HLA-A*0201–A**Q**WGPDPAAA (A2-AQW) crystals were grown in 0.2 M sodium malonate, 0.1 M BIS TRIS propane (pH 6.5), 20% PEG3350; HLA-A*0201–**RQF**GPD**WIV**A (A2-RQF[A]) crystals were grown in 0.2 M sodium sulphate, 0.1 M BIS TRIS propane (pH 6.5), 20% PEG 3350; HLA-A*0201–**RQ**WGPDPAA**V** (A2-RQW) crystals were grown in 0.1 M TRIS (pH 8), 20% PEG 8000, 15% glycerol; HLA-A*0201–**YQF**GPD**FPTA** (A2-YQF) crystals were grown in 0.1 M TRIS (pH 8), 25% PEG 4000, 15% glycerol; HLA-A*0201–**RQF**GPD**FPTI** (A2-RQF[I]) crystals were grown in 0.2 M potassium/sodium tartrate, 0.1 M BIS TRIS propane (pH 8.5), 20% PEG 3350; 1E6-A2-MVW crystals were grown in 0.1 M HEPES (pH 7.5), 15% PEG 4000, 0.2 M sodium acetate; 1E6-A2-YLG crystals were grown in 0.1 M sodium cacodylate (pH 6.5), 15% PEG 4000, 0.2 M sodium acetate; 1E6-A2-AQW crystals were grown in 0.2 M sodium citrate, 0.1 M BIS TRIS propane (pH 6.5), 20% PEG 3350; 1E6-A2-RQF(A) crystals were grown in 0.1 M HEPES (pH 7), 15% PEG 4000, 0.2 M sodium acetate; 1E6-A2-RQW crystals were grown in 0.2 M sodium cholride, 0.1 M MES (pH 6), 20% PEG 6000; 1E6-A2-YQF crystals were grown in 0.2 M sodium cholride, 0.1 M HEPES (pH 7), 20% PEG 3350; and 1E6-A2-RQF(I) crystals were grown in 0.1 M HEPES (pH 7.5), 15% PEG 4000, 0.2 M sodium acetate. Crystallization screens were conducted using an Art-Robbins Phoenix dispensing robot (Alpha Biotech Ltd.), and data were collected at 100 K at the DLS at a wavelength of 0.98 Å using an ADSC Q315 CCD detector. Reflection intensities were estimated using XIA2 (75), and the data were analyzed with Scala and the CCP4 package (76). Structures were solved with molecular replacement using Phaser (77). Sequences were adjusted with Coot (78), and the models were refined with REFMAC5. Graphical representations were prepared with PyMOL (79). The reflection data and final model coordinates were deposited with the PDB database (A2-MVW PDB: 5C0H; A2-YLG PDB: 5C0G; A2-AQW PDB: 5C0D; A2-RQF[A] PDB: 5C0J; A2-RQW PDB: 5C0F; A2-YQF PDB: 5C0E; A2-RQF[I] PDB: 5C0I; 1E6-A2-MVW PDB: 5C0A; 1E6-A2-YLG PDB: 5C09; 1E6-A2-AQW PDB: 5HYJ; 1E6-A2-RQF[A] PDB: 5C0C; 1E6-A2-RQW PDB: 5C08; 1E6-A2-YQF PDB: 5C07; and 1E6-A2-RQF[I] PDB: 5C0B).

### Peptide motif predictions.

Peptide motif predictions were performed by searching a viral database compiled using publicly available protein sequences of over 1,924,572 unique decamer peptides from the proteome of viral pathogens (80). The motif xOxGPDxxxO — where O is anyone of the hydrophobic amino acid residues A,V, I, L, M, Y, F, and W that might allow binding to HLA-A*0201 — was used as the search parameter.

### Statistics.

Pearson’s correlation analysis was performed to determine the relationship between TCR binding affinity and antigen potency, structural correlates, or thermodynamics using Origin Lab 9.0 pro.

## Author contributions

AMB, GD, AJS, BS, WR, AT, PJ, AF, AS, JJM, LW, PJR, and DKC performed experiments and analyzed the data. AKS, JR, CZ, JJM, MP, and DKC wrote the manuscript. AKS and DKC conceived and directed the study. AKS and DKC funded the study. All authors contributed to discussions.

## Supplementary Material

Supplemental data

## Figures and Tables

**Table 2:**
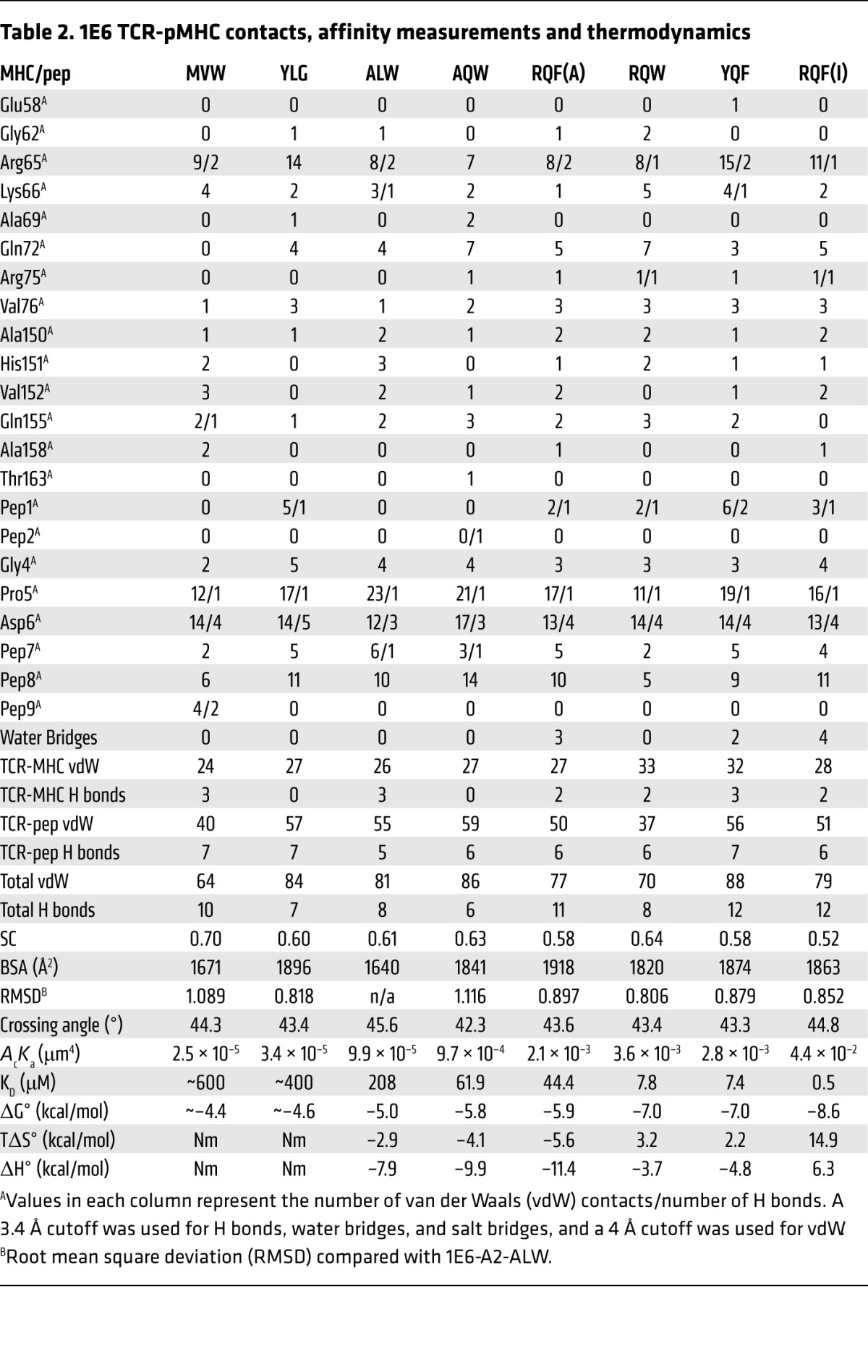
1E6 TCR-pMHC contacts, affinity measurements and thermodynamics

**Table 1:**
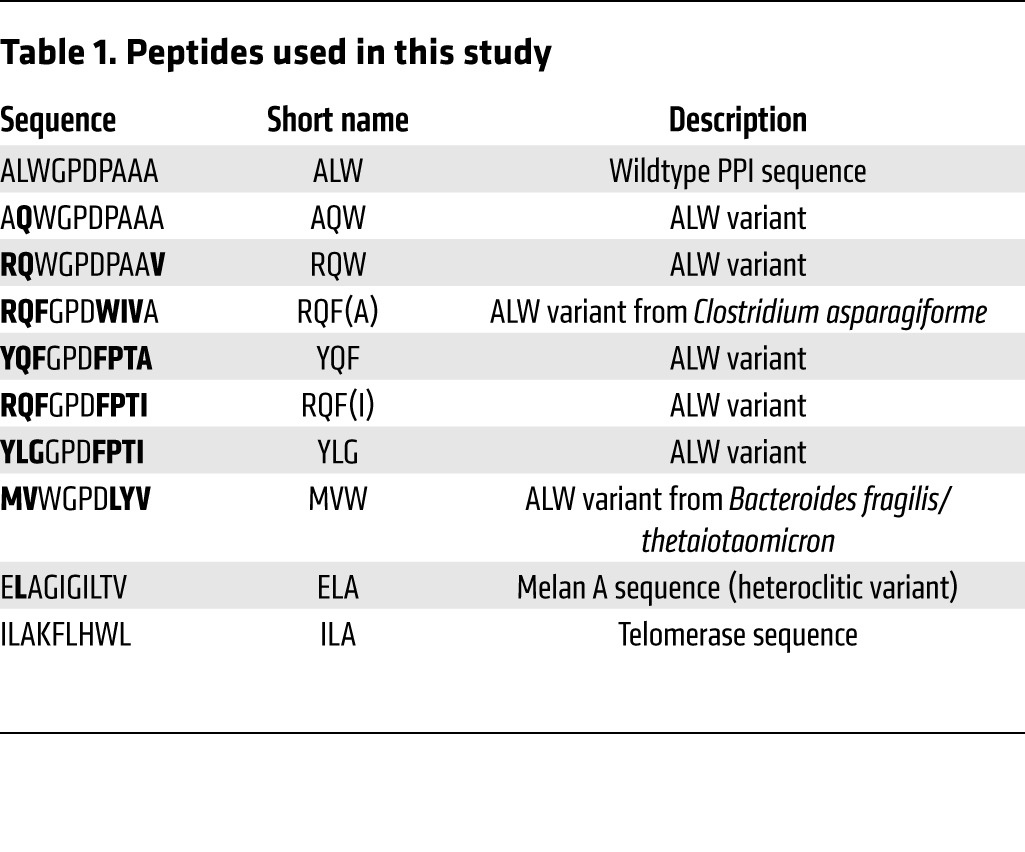
Peptides used in this study
