# Supplementary material for: Hotspot autoimmune T cell receptor binding underlies pathogen and insulin peptide cross-reactivity
Source: J Clin Invest. 2016 May 16;126(6):2191–204. doi: 10.1172/JCI85679 (PMC4887163; doi:10.1172/JCI85679)
Supplement: Supplemental data [file JCI85679.sd.pdf]

Supplementary Table S1. Data collection and refinement statistics for TCR-pMHC complex structures

	1E6-A2-MVW	1E6-A2-YLG	1E6-A2-AQW	1E6-A2-RQF(A)	1E6-A2-RQW	1E6-A2-YQF	1E6-A2-RQF(I)
**PDB code**	5C0A	5C09	5HYJ	5C0C	5C08	5C07	5C0B
**Data collection**							
Space group	P1	P1	P1	P1	P1	P1	P1
**Cell dimensions**							
a, *b*, *c* (Å)	43.9, 100.0, 123.3	43.8, 100.1, 122.4	41.1, 95.6, 119.4	43.9, 100.4, 122.7	43.8, 99.3, 122.2	43.7, 100.5, 122.1	43.9, 100.3, 122.4
α, β, γ (°)	96.7, 98.6, 95.8	96.9, 98.3, 96.5	81.1, 81.3, 85.4	96.9, 98, 96.1	96.3, 98.1, 96.4	97, 98.1, 96.6	97, 98.1, 96.5
Resolution (Å)	2.5	2.5	3.0	2.0	2.3	2.1	2.0
R_{merge} (%)	8.8	10.5	9.6	6.8	4.1	4.9	5
$I / \sigma I$	4.7	4.9	6.8	8.3	12.7	11.7	8
Completeness (%)	97.7	98.2	96.9	97.4	97.4	97	97.6
Redundancy	1.9	2	2.2	2	2.2	2.2	2
**Refinement**							
Resolution (Å)	2.5	2.5	3.0	2.0	2.3	2.1	2.0
No. reflections	68,869	67,353	32,310	132,894	79,156	107,577	121,919
No reflections in Rfree set	3,655	3,581	1,726	7,029	4,159	5,674	6,446
R_{work}/R_{free} (%)	19.3/ 23.2	20.7/ 25.9	21.2/ 29.4	18.9/ 22.7	20.6/ 27.2	19.1/ 23.7	21.4/ 26.8
**R.m.s. deviations**							
Bond lengths (Å)	0.01	0.01	0.01	0.02	0.02	0.02	0.02
Bond Angles (°)	1.71	1.74	1.52	2.04	1.91	1.92	1.86
Mean B value (Å^2)	68.0	63.3	68.3	52.3	55.3	50.6	45.6
Overall coordinate error (Å)	0.21	0.24	0.57	0.12	0.22	0.15	0.15

* One crystal was used for solving each structure.

Supplementary Table S2. Data collection and refinement statistics for pMHC structures

	A2-MVW	A2-YLG	A2-AQW	A2-RQF(A)	A2-RQW	A2-YQF	A2-RQF(I)
**PDB code**	5C0H	5C0G	5C0D	5C0J	5C0F	5C0E	5C0I
**Data collection**							
Space group	P1 21 1	P1 21 1	P1 21 1	P1 21 1	P1 21 1	P1 21 1	P1 21 1
**Cell dimensions**							
a, *b*, *c* (Å)	52.2, 79.5, 58.2	55, 79.1, 58.3	52.8, 81.2, 56.2	56.3, 80.1, 57	56.4, 79.8, 57	56.1, 79.7, 57	55.7, 79.7, 58.1
α, β, γ (°)	90, 115.7, 90	90, 115.6, 90	90, 112.3, 90	90, 115.8, 90	90, 115.7, 90	90, 115.7, 90	90, 115.7, 90
Resolution (Å)	1.4	1.4	1.7	1.6	1.5	1.5	1.5
R_{merge} (%)	4	4.1	8.4	5.1	4.9	8.5	4.6
$I / \sigma I$	11.9	14.4	8.7	11.7	14.7	12.2	12.1
Completeness (%)	98.3	94.6	98.8	99.6	99.4	99	99
Redundancy	3.5	3.3	3.9	3.6	4	3.9	3.7
**Refinement**							
Resolution (Å)	1.39	1.37	1.68	1.64	1.46	1.49	1.53
No. reflections	84,676	86,810	46,848	52,281	73,875	69,115	64,507
No reflections in Rfree set	4,467	4,579	2,496	2,796	3,913	3,669	3,448
R_{work}/R_{free} (%)	17.2/ 20.1	16.2/ 18.8	17.3/ 21.4	18.0/ 21.4	15.4/ 18.1	15.7/ 17.9	15.9/ 19.1
**R.m.s. deviations**							
Bond lengths (Å)	0.024	0.021	0.018	0.020	0.019	0.021	0.021
Bond Angles (°)	2.28	2.24	1.83	1.96	2.09	2.36	2.07
Mean B value (Å^2)	22.5	19.3	20.4	26.9	18.3	19.6	25.2
Overall coordinate error (Å)	0.043	0.042	0.086	0.072	0.045	0.049	0.058

* One crystal was used for solving each structure.

Supplementary Table 3: Affinity measurements, using surface plasmon resonance, for the 1E6 TCR binding to peptide variants at different temperatures.

Temp (ºC)	ALW	RQW	AQW	RQF(A)	YQF	RQF(I)
5	185.5	8.2	56.3	34.7	5.4	0.9
13	168.2	7.3	50.8	32.4	5.3	1
18	176.4	7.2	52	36.7	6	0.87
25	208	7.8	61.9	44.4	7.4	0.49
32	297.5	9.5	113.8	89	9	0.38
37	482.4	11.8	231	107.2	10.5	0.42

Values in the table are affinities calculated from equilibrium binding experiments at different temperatures, K_D (µM)

Supplementary Table 4: Peptide sequences from the known viral proteome containing a xOxGPDxxxO motif (O=hydrophobic amino acid). 53 peptide motifs identified in 151 different sequences out of 10733 total sequences (1.4%).

PEPTIDE	PROTEIN [VIRUS]
LILGPDDHVL	RABVR GLYCOPROTEIN G [RABIES VIRUS CHINA/MRV]
IILGPDGHVL	VGLG_RABVT GLYCOPROTEIN G [RABIES VIRUS ALGERIA/1991]
RLPGPDTRHL	A36R [MONKEYPOX VIRUS ZAIRE-96-I-16]
IILGPDGNVL	VGLG_RABVB GLYCOPROTEIN G [RABIES VIRUS SILVER-HAIRED BAT-ASSOCIATED]
GVTGPDSKAV	NEURAMINIDASE [INFLUENZA A VIRUS]
HMGGPDDPAV	TEGUMENT PROTEIN VP13/14 [MACACINE HERPESVIRUS 1]
VVRGPDCGGM	POLYPROTEIN PRECURSOR [SAPOVIRUS MC10]
KVQGPDLRDV	POLYPROTEIN [HUMAN COSAVIRUS B]
KISGPDLLNA	DBP [HUMAN ADENOVIRUS 1]
PVSGPDYPPL	BFRF2 [HUMAN HERPESVIRUS 4 TYPE 2]
SLPGPDAEKW	MAJOR CORE PROTEIN LAMBDA 1 [MAMMALIAN ORTHOREOVIRUS 3]
QLPGPDVHPL	MC142R [MOLLUSCUM CONTAGIOSUM VIRUS SUBTYPE 1]
IIKGPDGHIL	VGLG_LBV GLYCOPROTEIN G [LAGOS BAT VIRUS]
GISGPDDEAV	NEURAMINIDASE [INFLUENZA A VIRUS USA:HUSTON/AA/1945 H1N1]
RVCGPDEKWI	NUCLEOPROTEIN [THOGOTO VIRUS]
GISGPDDGAV	NEURAMINIDASE [INFLUENZA A VIRUS LENINGRAD/1/1954 H1N1]
GITGPDATAV	NEURAMINIDASE [INFLUENZA A VIRUS]
GVDGPDNNAL	NEURAMINIDASE [INFLUENZA B VIRUS]
GVDGPDSNAL	NEURAMINIDASE [INFLUENZA B VIRUS (STRAIN B/SINGAPORE/222/1979]
MAHGPDMASV	DNA REPLICATION ORIGIN-BINDING HELICASE [SUID HERPESVIRUS 1]
GISGPDNEAV	NEURAMINIDASE [INFLUENZA A VIRUS USA:PHILA/1935 H1N1]
GISGPDNGAV	NEURAMINIDASE [INFLUENZA A VIRUS CHICKEN/SHANTOU/4231/2003 H5N1]
AISGPDNGAV	NEURAMINIDASE [INFLUENZA A VIRUS CHICKEN/HONG KONG/37.4/2002 H5N1]
LLQGPDGSIY	PROTEIN DR1 [HUMAN HERPESVIRUS 7]
GIPGPDYDFV	MC080R [MOLLUSCUM CONTAGIOSUM VIRUS SUBTYPE 1]
GVDGPDNDAL	NEURAMINIDASE [INFLUENZA B VIRUS]
GVDGPDSDAL	NEURAMINIDASE [INFLUENZA B VIRUS MARYLAND/1959]
IIKGPDGQIL	TRANSMEMBRANE GLYCOPROTEIN G [MOKOLA VIRUS]
YAPGPDGTII	IVA2 [HUMAN ADENOVIRUS 1]
SVWGPDGALA	EARLY TRANSCRIPTION FACTOR [PSEUDOCOWPOX VIRUS]
HAVGPDFRKY	NONSTRUCTURAL PROTEIN NSP3 [AURA VIRUS]
RLCGPDTAAW	TRANSCRIPTIONAL REGULATOR ICP4 [HUMAN HERPESVIRUS 1]
LAQGPDLRDL	POLYPROTEIN [HUMAN COSAVIRUS D]
LAVGPDDEVA	RIBONUCLEOTIDE REDUCTASE SUBUNIT 1 [HUMAN HERPESVIRUS 5]
TYQGPDDVYV	MINOR CORE PROTEIN [MAMMALIAN ORTHOREOVIRUS 3]
PYNGPDKKSL	3B [HUMAN COSAVIRUS B]
TWEGPDENAI	POLYPROTEIN [POWASSAN VIRUS]
SVPGPDPRLW	HHV6U UNCHARACTERIZED PROTEIN DR2 [HUMAN HERPESVIRUS 6A STRAIN UGANDA-1102]
TLEGPDPGRA	ENCAPSIDATION PROTEIN IVA2 [HUMAN MASTADENOVIRUS E]
EYYGPDYPYW	ORF1 [TORQUE TENO TADARIDA BRASILIENSIS VIRUS]
CFEGPDEHEI	POLG_YEFVC GENOME POLYPROTEIN [YELLOW FEVER VIRUS ISOLATE IVORY COAST/1999]
DATGPDGPLV	VP2 [GREAT ISLAND VIRUS]
EYIGPDLWPF	HELICASE-PRIMASE SUBUNIT [HUMAN HERPESVIRUS 6A]
CMVGPDYAYF	1AB POLYPROTEIN [MIDDLE EAST RESPIRATORY SYNDROME CORONAVIRUS]
NFCGPDGYPL	NSP2-PP1A/PP1AB [SARS CORONAVIRUS]
PAPGPDPLEI	EBNA-3B NUCLEAR PROTEIN [HUMAN HERPESVIRUS 4]
YFNGPDRDLW	HASV1 NON-STRUCTURAL POLYPROTEIN 1AB [HUMAN ASTROVIRUS-1]
AAGGPDDEPA	RIBONUCLEOTIDE REDUCTASE SUBUNIT 1 [PAPIINE HERPESVIRUS 2]
SFWGPDGCWY	POLYPROTEIN [WESSELSBRON VIRUS]
YFNGPDKDLW	HASV4 NON-STRUCTURAL POLYPROTEIN 1AB [HUMAN ASTROVIRUS-4]
AAGGPDQTAA	DNA PACKAGING TERMINASE SUBUNIT 2 [PAPIINE HERPESVIRUS 2]
TASGPDEREA	TEGUMENT PROTEIN VP13/14 [SUID HERPESVIRUS 1]
PAAGPDAVEA	ORF75 [HUMAN HERPESVIRUS 8]

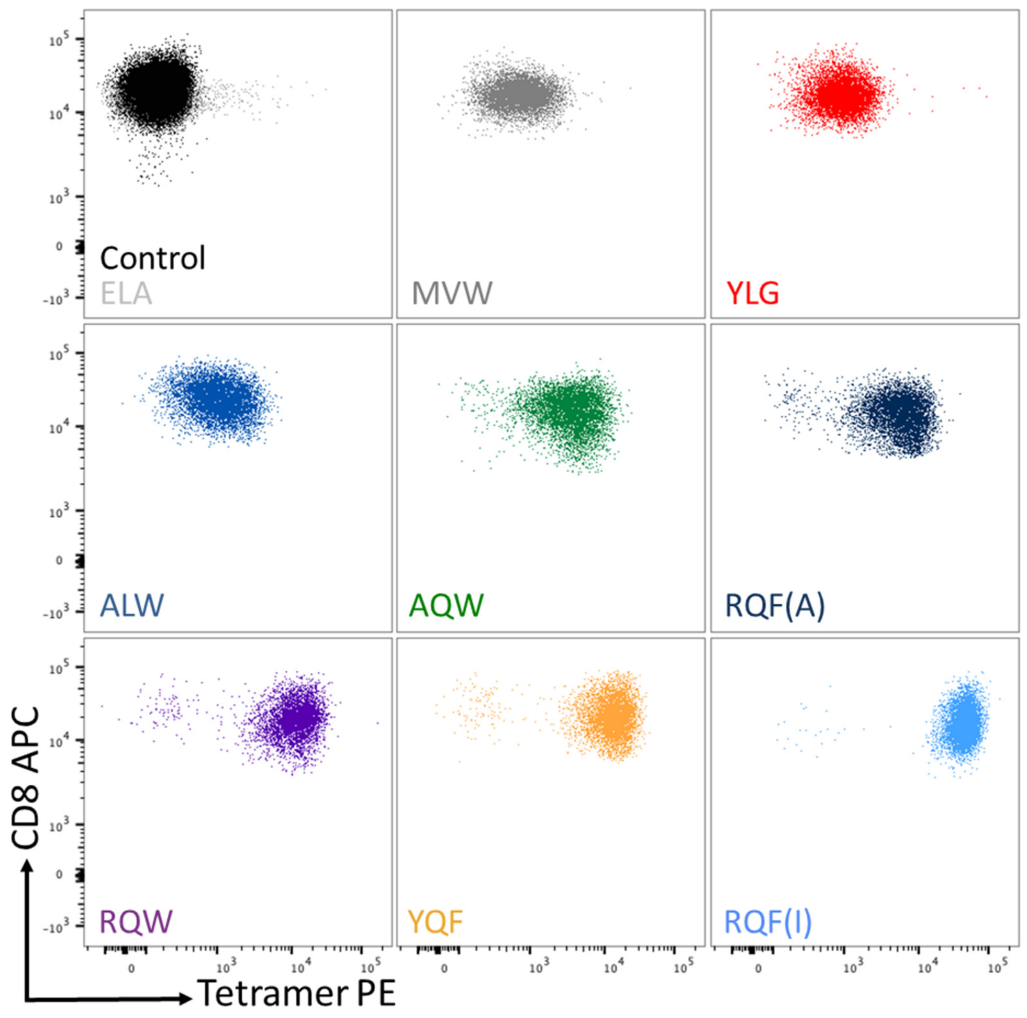

Supplementary Figure S1: The 1E6 clone was left unstained (control) or stained with PE-conjugated 1E6 APL or irrelevant (ELA) tetramers, as indicated. The dots plots were generated by sequentially gating based on forward and side scatter, then viability and CD8 expression. The MFI was established for the population shown without any further gating.
